# Altered circadian rhythm, sleep, and *rhodopsin 7*–dependent shade preference during diapause in *Drosophila melanogaster*

**DOI:** 10.1073/pnas.2400964121

**Published:** 2024-06-25

**Authors:** Geoff T. Meyerhof, Sreesankar Easwaran, Angela E. Bontempo, Craig Montell, Denise J. Montell

**Affiliations:** ^a^Department of Molecular, Cellular, and Developmental Biology, Santa Barbara, CA 93106; ^b^Neuroscience Research Institute, University of California, Santa Barbara, CA 93106

**Keywords:** *Drosophila melanogaster*, diapause, circadian rhythm, sleep

## Abstract

To survive adverse environments, many animals enter a dormant state such as hibernation, dauer, or diapause. Various *Drosophila* species undergo adult reproductive diapause in response to cool temperatures and/or short day-length. While flies are less active during diapause, it is unclear how adverse environmental conditions affect circadian rhythms and sleep. Here we show that in diapause-inducing cool temperatures, *Drosophila melanogaster* exhibit altered circadian activity profiles, including severely reduced morning activity and an advanced evening activity peak. Consequently, the flies have a single activity peak at a time similar to when nondiapausing flies take a siesta. Temperatures ≤15 °C, rather than photoperiod, primarily drive this behavior. At cool temperatures, flies rapidly enter a deep-sleep state that lacks the sleep cycles of flies at higher temperatures and require high levels of stimulation for arousal. Furthermore, we show that at 25 °C, flies prefer to siesta in the shade, a preference that is virtually eliminated at 10 °C. Resting in the shade is driven by an aversion to blue light that is sensed by Rhodopsin 7 outside of the eyes. Flies at 10 °C show neuronal markers of elevated sleep pressure, including increased expression of Bruchpilot and elevated Ca^2+^ in the R5 ellipsoid body neurons. Therefore, sleep pressure might overcome blue light aversion. Thus, at the same temperatures that cause reproductive arrest, preserve germline stem cells, and extend lifespan, *D. melanogaster* are prone to deep sleep and exhibit dramatically altered, yet rhythmic, daily activity patterns.

## Significance

Climate change is impacting many animals, including insects. In diverse organisms, adverse environments trigger dormancy programs such as hibernation and diapause. Fruit flies undergo diapause to survive winter. Here, using the methods that we developed, we show that the same cool temperatures that delay fruit fly reproduction and extend lifespan also promote deep sleep. Cool flies rapidly fall asleep and are difficult to arouse. Once awake, they immediately fall back to sleep. Whereas in warm environments, midday blue light drives flies to siesta in the shade, in cool temperatures the need to sleep overwhelms light aversion. Animals that adjust their behavior directly to temperature, rather than day length, may be more resilient to a changing climate.

As the climate changes, understanding organismal responses to temperature becomes more important. Dormancy programs such as hibernation, torpor, dauer, and diapause allow animals to endure adverse environments while extending lifespan and reproductive capacity (1–5). Diapause is a well-documented phenomenon in insect species including fruit flies as well as in animals such as killifish and mice (6, 7). Diapause enhances survival by allowing animals to delay aging, development, and/or reproduction until environmental conditions improve (8).

Various *Drosophila* species undergo adult reproductive diapause in response to cool temperatures and/or short day length (9–11). Although initially characterized as an arrest of ovarian development at the yolk-uptake stage, diapause is now recognized to be a complex program that affects nearly every aspect of life. Diapause entails arrest of growth and development, altered metabolism and behavior, and lifespan extension. To enter diapause, animals must first sense environmental change, and then adjust their behavior and physiology. Diapausing flies reduce food intake and overall activity; however, it is unknown whether this represents a cool-temperature-induced immobility or an actively modified behavioral program.

The molecular and cellular effects of diapause conditions on fly physiology, metabolism, reproduction, and lifespan are under active investigation (12–17). For example, cool temperatures rapidly dampen activity in circadian pacemaker neurons, reducing levels of secreted neuropeptides and hormones, leading to ovarian arrest (17, 18). So, sensing environmental conditions, primarily temperature, precedes and causes reproductive arrest.

Many open questions remain (8), including whether diapause affects circadian rhythms and sleep. Furthermore, although *Drosophila* sleep has been well characterized under optimal conditions, the effects of adverse environments on sleep are unclear. Here, we describe that at 10 to 15 °C, flies show dramatically altered circadian rhythms and sleep, which are independent of changes in juvenile hormone (JH). Thus, the same cool temperatures that induce reproductive arrest via decreased JH production, cause JH-independent behavioral effects.

## Results

### Diapause-Inducing Conditions Reshape the *Drosophila* Circadian Activity Profile.

**Fig. 1.**
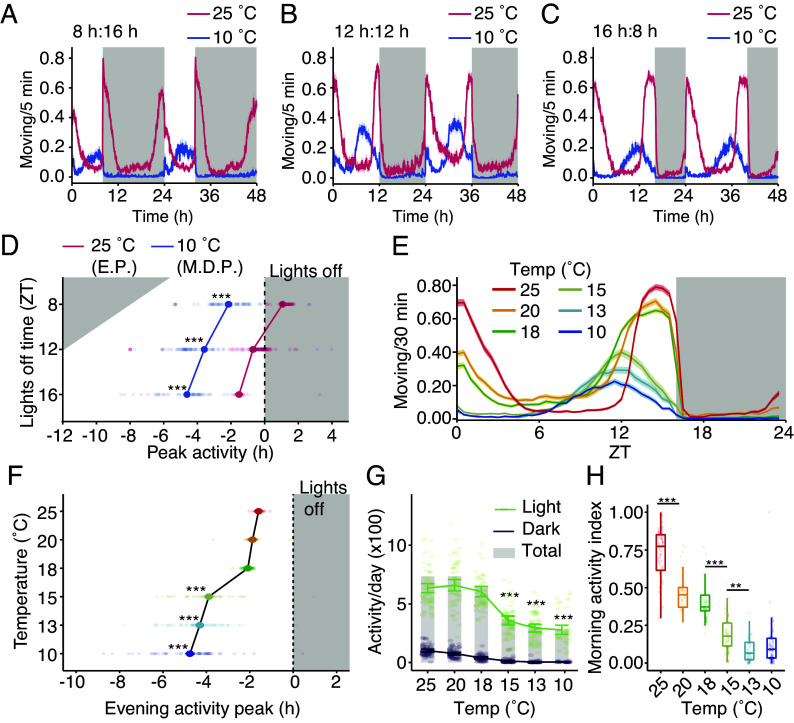
Diapause shifts activity pattern from crepuscular to midday peak. (*A–C*) 48-h average actograms of flies under the indicated conditions (*A*) 25 °C (red, n = 63 flies) or 10 °C (blue, n = 46). (*B*) 25 °C (red, n = 74) or 10 °C (blue, n = 55). (*C*) 25 °C (red, n = 64) or 10 °C (blue, n = 51). (*D*) Time of peak activity vs. photoperiod from panels *A*–*C*. At 25 °C (E.P., evening peak time); at 10 °C (M.D.P., midday peak time). Two-way ANOVA [temperature (t), photoperiod (p), and t:p interaction] with the Tukey HSD test. (*E*) Effect of temperature on circadian activity profiles of flies at the indicated temperatures. n = 51 to 64 flies/temp. (*F*) Effect of temperature on the evening activity peak for flies in *E*. One-way ANOVA with the factor of temperature with Tukey HSD tests. Significance determined vs. the 25 °C group. (*G*) Daytime (light), nighttime (dark), and total activity (gray bar) of flies shown in *E*. Two-way ANOVA with factors of time (Light and Dark) and temperature. Tukey HSD test. Significance indicates activity in the light compared to the 25 °C group. (*H*) Morning activity index (proportion of activity between ZT0 and ZT5) vs. temperature for flies shown in *E*. One-way ANOVA followed by Tukey HSD test to calculate individual group differences. Means ± SEMs. ***P < 0.001, *P < 0.01.

To determine the effect of diapause-inducing conditions [10 °C and 8 h light (L) and 16 h dark (D) cycles (8L:16D)] on circadian activity, we first employed the *Drosophila* Activity Monitor (DAM) system to record the movement of flies throughout the day (19, 20). In this assay, flies are individually housed in tubes with access to a sucrose food source. Their activity is recorded via an infrared sensor running through the center of each tube. We allowed female flies to acclimatize to either 25 °C or 10 °C for ≥ 24 h under varying LD cycles and then recorded their activity for four days. At 25 °C in 8L:16D, the flies displayed prominent morning and evening activity peaks, which flanked a period of inactivity in the middle of the day known as the siesta (Fig. 1*A*, red traces) (21). In this short photoperiod condition (8L:16D), the evening activity peaked at lights off, while their morning activity peak preceded lights on, and is referred to as morning anticipation. Thus, the majority of their activity occurred at night (Fig. 1*A*), consistent with a previous report (22).

When flies were maintained at 10 °C under an identical short photoperiod, their activity profile was considerably altered (Fig. 1*A*). In addition to reducing overall activity, diapausing conditions markedly reduced the morning activity peak and advanced the evening peak (Fig. 1*A*). To determine whether the residual morning activity was simply a startle response due to the light turning on, we repeated the experiment with a ramping light. In this paradigm, the light intensity gradually increased to 400 lx from ZT 0 to 6 and gradually decreased to 0 lx from ZT 6 to 12. Under ramping light, flies at 25 °C retained a pronounced morning activity peak, including morning anticipation (*SI Appendix*, Fig. S1*A*). In contrast, flies at 10 °C showed little to no morning activity and no morning anticipation (*SI Appendix*, Fig. S1*B*). Thus, at 10 °C, morning activity and the midday siesta are eliminated, leaving a single activity peak.

To distinguish between the effects of temperature and photoperiod, we recorded the activity of flies maintained at 10 °C with a longer day. When we extended the photoperiod to either 12L:12D or 16L:8D, flies at 10 °C, still exhibited a single activity peak during the mid to late day (Fig. 1 *A*–*D*). These data demonstrate that it is the cool temperature rather than photoperiod that dictates the activity pattern. In contrast, at 25 °C the phase of the flies’ evening activity peak was much closer to the L:D transition. The evening activity peak occurred just after lights off in flies maintained at 25 °C and 8L:16D (Fig. 1 *A* and *D*), whereas under a longer photoperiod, the evening activity shifted to before lights off, reaching its zenith 1.5 h before nighttime (Fig. 1 *B*–*D*).

We wondered whether the activity profile would change gradually with temperature, or, alternatively, whether there would be an abrupt change at diapause-inducing temperatures. To test this, we subjected flies to two additional cool temperatures (13 °C and 15 °C) as well as 20 °C and 18 °C, which still support crepuscular activity (peaks near dawn and dusk). For these experiments, we housed wild-type flies under 16L:8D cycles, as an extended light period helped to disambiguate morning vs. evening activity onset. As the temperature decreased, the evening activity peak shifted to earlier times (Fig. 1 *E* and *F*), total activity decreased (Fig. 1*G*), and the morning activity index (the proportion of daytime activity occurring from ZT 0 to 6) diminished (Fig. 1*H*). Notably, the largest changes in all parameters occurred from 18 °C to 15 °C (Fig. 1 *E*–*H*). In addition, 15 °C is also the temperature below which egg production ceases (17), so these results show that circadian activity responds to the same temperatures that induce reproductive arrest. These results are consistent with recent studies that show that reproductive arrest and recovery are more dependent on temperature than photoperiod (13, 17, 18).

To test the speed at which flies are able to adapt their rhythms from diapause to nondiapause conditions (and vice versa), we recorded the circadian activity of flies for three full days at either 25 °C or 10 °C. In the middle of day four (ZT 6), we rapidly changed the temperature from either 25 °C to 10 °C or 10 °C to 25 °C (*SI Appendix*, Fig. S1*C*). This change immediately affected the flies’ activity rhythms. Flies shifted from 10 °C to 25 °C increased their activity and, strikingly, in less than 12 h adopted an evening activity peak that was aligned with the L:D transition. Flies shifted from 25 °C to 10 °C rapidly reduced overall activity, suggesting a direct response to cool temperature. However, the advancement of their evening activity peak was not apparent until their first full day at 10 °C.

### Cool Temperature Diminishes Aversion to Sleeping in the Light.

A key feature of the diapause activity rhythm is the advanced timing of the evening activity peak, which initiates around midday (Fig. 1 *A*–*D*). Concomitant with this increased midday activity is a reduction in the daytime siesta. The duration of the daytime siesta is known to be positively correlated with light intensity and temperature (23, 24), so the daytime siesta may reflect a light avoidance behavior, possibly to mitigate desiccation during hot summer days. Therefore, we wondered whether diapause-inducing temperatures might also reduce aversion to light.

**Fig. 2.**
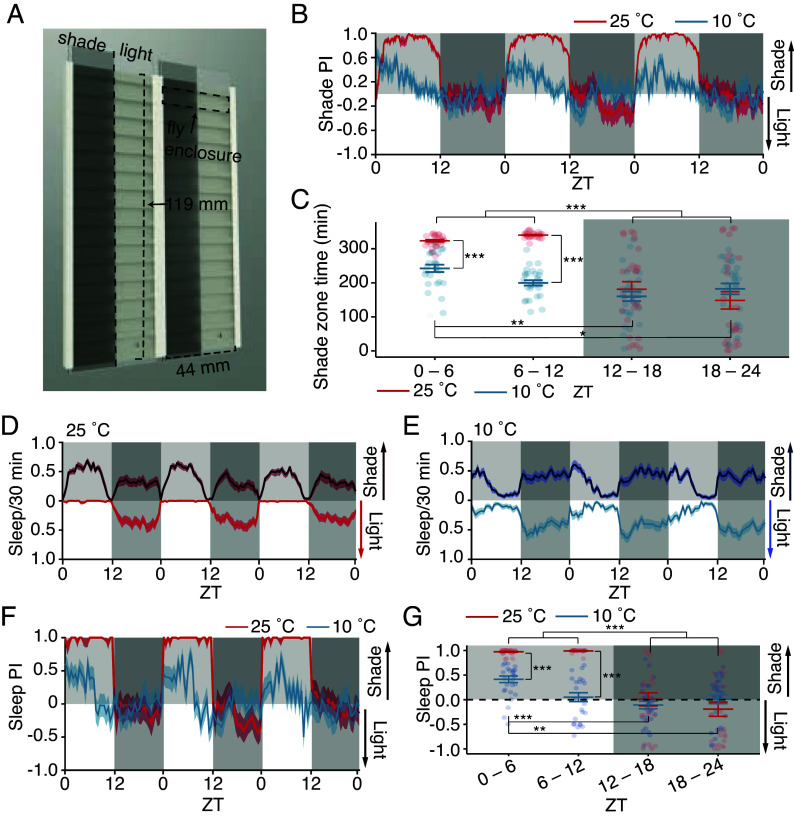
Cool temperature suppresses aversion to light. (*A*) Behavioral arena used in shade preference assays (*Materials and Methods*). (*B*) Shade preference index (PI) of flies over three days at 25 °C (red) or 10 °C (blue). The plots represent mean shade PI ± SEM (see *Materials and Methods* for formula). (*C*) Average time in shade over 3-d during the first and second half of daytime and nighttime. Error bars, SEM. Data were analyzed by aligned-rank transform two-way ANOVA, examining factors of time, temperature, and time:temperature interaction. Individual differences between groups were analyzed by aligned-rank transform contrast, with the Bonferroni-adjusted *P* value. (*D* and *E*) Sleep location for 3 d from flies housed at 25 °C (D, red) or 10 °C (E, blue). (*F*) Sleep PI (see *Materials and Methods* for formula) from flies housed at 25 °C (red) or 10 °C (blue) for three days. In A, *B*, *D*, *E*, and *F*, values > 0 indicate preference for the shaded zone, and values < 0 indicate a preference for the unshaded zone. Error bars represent SEM. (*G*) Average sleep preference for flies at 25 °C (red) or 10 °C (blue) during the early or late daytime and nighttime. Aligned-rank transform two-way ANOVA, examining factors of time, temperature, and time:temperature interaction. Individual differences between groups analyzed by aligned-rank transform contrast with Bonferroni multiple testing correction. *B*–*G*: n = 27 to 29 Canton S (wild-type flies) per condition, under a 12 h light:12 h dark cycle. ***P < 0.001.

To test the impact of temperature on the preference between shade vs. bright light, we designed a custom behavioral arena. We housed 30 flies individually, each in a 44 mm × 6 mm enclosure with constant access to a 5% sucrose food source (Fig. 2*A*). We placed a neutral density filter along one-half of each enclosure, so that flies could choose between spending time in a shaded zone (180 lx) or directly in brighter light (1,700 lx; *SI Appendix*, Fig. S1*D*). Except for the initial 0.75 °C increase in temperature when the lights turned on (ZT 0), the temperature in the arena did not increase further during the rest of the day (ZT 1 to 12), regardless of whether it was warm (*SI Appendix*, Fig. S1*E*) or cool (*SI Appendix*, Fig. S1*F*). Moreover, the temperatures of the shaded and unshaded zones were virtually identical at all times (*SI Appendix*, Fig. S1 *E* and *F*). To provide a static light source for real-time video tracking, we backlit the arena with a near-infrared (IR) light-emitting diode (LED) (850 nm), which is imperceptible to flies.

We found that maintaining flies at 10 °C reduced their preference for a shady environment. At 25 °C, flies ardently preferred spending time in the shaded zone during the day (Fig. 2 *B* and *C*). Consistent with previous reports (24, 25), we found that, during the daytime, the flies’ preference for shade was highly correlated with the length of their immobility (*SI Appendix*, Fig. 1 *G*, *Left*), indicating that they rest in the shade. In contrast, flies at 10 °C displayed a marked reduction in their preference for shade (Fig. 2 *B* and *C*). Unlike flies at 25 °C, which showed a negative correlation between how far they moved vs. time spent in the shade (*SI Appendix,* Fig. S1 *G*, *Left*), at 10 °C, there was no correlation between these two parameters (*SI Appendix,* Fig. S1 *G*, *Right*), indicating that flies at 10 °C were equally prone to rest under high or low illumination.

We next analyzed flies’ preference for sleeping in the shade, using the typical definition of sleep as five consecutive minutes of inactivity (26–28). *Drosophila* daytime sleep consists of short, light sleep bouts that peak around the middle of the day (29). Compared to flies at 25 °C, those maintained at 10 °C exhibited a similar total amount of daytime sleep, although the timing was markedly altered such that they slept late into the morning and were more active in the afternoon (*SI Appendix*, Fig. S1 *H* and *I*). Additionally, nighttime sleep was substantially increased at 10 °C, with these flies sleeping nearly all night (*SI Appendix*, Fig. S1 *H* and *I*). At 25 °C, nearly all of the flies’ daytime sleep occurred in the shade (Fig. 2*D*) (30, 31). In contrast, flies at 10 °C showed a reduced preference for shaded daytime sleep during the morning (ZT 0 to 6) and no preference for shade in the evening (ZT 6 to 12) (Fig. 2 *E–G*). In total, 10 °C markedly reduced aversion to sleeping under bright illumination (Fig. 2*G*).

Diapause is a holistic program of changes to fly behavior, physiology, and reproductive development that initiates in response to changes in the environment, primarily temperature. One hallmark of diapause is the arrest of yolk-uptake during oogenesis, which results in small, underdeveloped ovaries (*SI Appendix*, Fig. S2 *A* and *B*) (12). Ovarian arrest during diapause is in part regulated by a reduction in JH signaling (8, 32, 33), which is regulated by activity of circadian neurons. Treating flies with the JH analog methoprene partially reverses this arrest, enlarging the ovaries (*SI Appendix*, Fig. S2 *B* and *C*) (13, 34). To test whether the alterations we observed in flies’ circadian rhythm and sleep were also regulated by JH, we added 100 µg of methoprene to each of the fly enclosures and recorded their activity, sleep, and preference for shade. Adding methoprene had almost no impact on activity (*SI Appendix*, Fig. S2*D*), sleep (*SI Appendix*, Fig. S2*E*), or preference for shaded sleep (*SI Appendix*, Fig. S2*F*). This indicates that the effect of temperature on preference for shaded sleep is either upstream of JH, as are temperature-dependent changes in circadian neuron activity, or possibly parallel to/independent of the JH pathway.

### At Diapause-Inducing Temperatures, Flies Sleep Deeply.

**Fig. 3.**
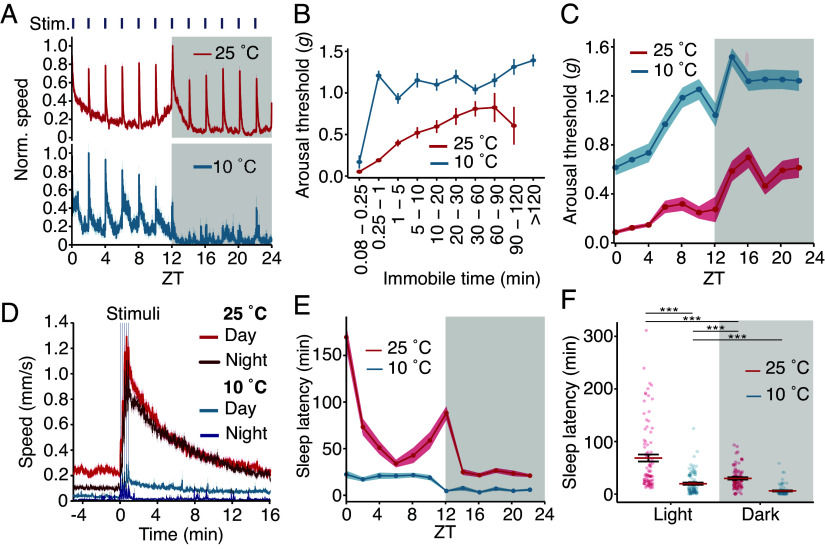
Diapause induces rapid-onset deep sleep. (*A*) Activity profile of flies housed at 25 °C (red) or 10 °C (blue) subjected to vibration once every 2 h over the span of 1 d. Data binned each minute and normalized to peak speed. (*B*) The *Y* axis displays average arousal threshold (vibration intensity in *g* force) required to induce locomotion in flies immobile for varying lengths of time. At 25 °C, no flies were immobile for > 120 min. Means ± SEMs. (*C*) Mean *g* force required to induce locomotion in quiescent flies at 10 °C or 25 °C, over 1 d. (*A*–*G*) Data represent 1,959 responses from 179 *w^1118^* flies. (*D*) Average walking speed of flies in response to vibration at 25° or 10 °C during the day (ZT 0 to 10) and night (ZT 12 to 22). Time 0 denotes the start of the stimulus train, with the vertical lines indicating each stimulus. (*E*) Circadian sleep latency (average time to sleep for immobile flies that responded) of flies housed at 25 °C (red) or 10 °C (blue). Shading represents SEMs. (*F*) Quantification of sleep latency during daytime (light) and nighttime (dark) from flies at 25 °C or 10 °C. n = 89 to 90 flies/temperature. Aligned-rank transform two-way ANOVA, examining factors of light, temperature (*P* < 0.001), and light:temp interaction. Individual differences between groups were analyzed by aligned-rank transform contrast with a Bonferroni multiple testing comparison. ***P < 0.001.

A defining characteristic of sleep, as opposed to simple immobility, is decreased sensitivity to external stimuli (35), so we compared the arousal thresholds of flies at 25 °C and at 10 °C. We used vibrating motors to deliver a set of five gradually increasing vibration stimuli, each 3 s in length, once every 2 h during a 1 d recording (Fig. 3*A* and *SI Appendix*, Fig. S3 *A*–*D*). We measured movement via real-time video tracking capable of resolving movements of less than one body-length (~3 mm; Movie S1) and scored arousal as the vibration required to induce 3 mm of movement in flies that were immobile prior to the start of the stimulus. We observed that at 25 °C, flies responded to vibration by transiently moving both during the day and at night (Fig. 3*A*, red line). Similar to flies at 25 °C, flies at 10 °C, moved in response to vibration during the day (Fig. 3*A*, blue line). However, the stimuli had a markedly smaller effect on flies at 10 °C during the night, suggesting again that these flies were in a deep sleep. Despite the vibrations transiently increasing activity and reducing sleep (Fig. 3*A* and *SI Appendix*, Fig. S3*E*), there was no overt effect of vibration on flies’ shaded sleep preference index, indicating that this preference is unchanged by wake-promoting stimuli (*SI Appendix,* Fig. S3*F*).

Sleep depth (arousal threshold) in *Drosophila* changes with the length of their immobility, suggesting that as in mammals, fly sleep may also have stages, and therefore architecture (i.e., distinct periods of sleep characterized by different arousal thresholds) (35–37). To assess whether diapause impacts sleep architecture, we analyzed how arousal threshold changed with the length of immobility. Flies maintained at 10 °C were on average immobile for ~10-fold longer than flies at 25 °C prior to the onset of the stimuli (*SI Appendix*, Fig. S3*G*). At 25 °C, the arousal threshold, which is the vibration intensity (in units of gravitational force, *g*), increased greatly if the flies were immobile for over 5 min. After 5 to 15 s of immobility, nondiapausing flies could be aroused easily with a low intensity (Fig. 3*B*). After 5 to 10 min, 10-fold more *g* force was required to stir the flies (Fig. 3*B*). The force required peaked at 60 to 90 min of immobility (Fig. 3*B*), after which it decreased (Fig. 3*B* and *SI Appendix*, Fig. S3*H*).

The arousal threshold of flies at 10 °C exhibited a different pattern. Maintaining flies at 10 °C did not simply impair their ability to sense vibration because flies that were immobile for 5 to 15 s had a low arousal threshold similar to flies at 25 °C (Fig. 3*B*). However, at immobilities exceeding 15 s, the arousal threshold of diapausing flies rapidly increased. After only 0.25 to 1.0 min of immobility, the arousal threshold of diapausing flies exceeded even the greatest value that we observed in flies at 25 °C (Fig. 3*B*). Also, unlike flies at 25 °C, the arousal threshold of diapausing flies did not peak after 60 to 90 min of immobility, but remained elevated, even in flies that had been immobile for > 2 h (Fig. 3*B*). Thus, diapausing flies rapidly initiate a long-lasting, deep-sleep state that is distinct from nondiapause sleep.

### Flies Awakened in Diapause Rapidly Resume Sleep.

To assess the circadian influence on sleep depth, we analyzed how the arousal threshold changed throughout the day at 25 °C and 10 °C. During the day, flies at 25 °C had their highest arousal threshold (i.e., deepest sleep) during the period of their midday siesta (Fig. 3*C*) and a higher arousal threshold at night (Fig. 3*C*). In contrast, the daytime arousal threshold of flies at 10 °C started out sixfold higher and further increased during the day, with their deepest sleep occurring at ~ZT 10 (Fig. 3*C*). Unlike flies at 25 °C, the proportion of diapausing flies responding to vibration stimuli displayed a nearly monotonic decrease as the day progressed (*SI Appendix*, Fig. S3*I*), suggesting that the cool temperature rapidly increased sleep pressure throughout the day. However, similar to flies at 25 °C, flies at 10 °C had a higher arousal threshold and lower proportion of responders at night compared to the day (Fig. 3*C* and *SI Appendix*, Fig. S3*I*). Overall, flies at 10 °C exhibited rhythmic circadian sleep patterns, but they were profoundly different from those of flies at 25 °C.

We reasoned that if the cool temperature increased sleep pressure, then there would be differences in how quickly diapausing and nondiapausing flies resume sleep after having been startled by the vibration stimulus. To address this, we plotted how walking speed changed in response to vibration. The average walking speed prior to a vibration stimulus was lower during the night (ZT 12 to 22) than during the day (ZT 0 to 12) at both 25 °C and 10 °C (Fig. 3*D* and *SI Appendix*, Fig. S3*J*). Upon vibration, flies at 25 °C, on average, rapidly increased their walking speed (0.23 mm/s day; 0.10 mm/s night) to a maximum of 1.10 mm/s during the night and 1.29 mm/s during the day (Fig. 3*D* and *SI Appendix*, Fig. S3*J*). After the stimuli, their average daytime and nighttime walking speed gradually decayed back to the baseline at roughly the same rate over 16 min (*SI Appendix*, Fig. S3*J*). Flies at 10 °C also increased their walking speed in response to the vibration stimuli; however, during the nighttime, diapausing flies rapidly stopped moving (Fig. 3*D* and *SI Appendix F*ig. S3*J*).

To quantify this effect further, we recorded sleep latency for diapausing and nondiapausing conditions. We defined sleep latency as the time it takes for immobile flies that are responsive to the vibration stimuli to initiate an extended bout of rest (5 consecutive minutes of immobility). The sleep latency of flies at 25 °C dramatically changed throughout the day, with their longest sleep latency occurring in the morning and early night (Fig. 3*E*), and their shortest daytime latency occurring at midday (ZT 6) (Fig. 3*E*). Late in the night (ZT 14 to 22), the sleep latency of flies at 25 °C was more consistent, averaging ~25 min. In contrast, the sleep latency of diapausing flies was markedly reduced, both during the daytime and nighttime (Fig. 3*E*). The sleep latency of flies at 10 °C fluctuated little over the course of the day or night (Fig. 3 *E* and *F*); however, it was still higher during the day compared to the night, showing again that the flies maintained rhythmic behavior. These data demonstrate that flies in diapause rapidly resume sleep upon being startled. In total, the arousal threshold data reveal that diapausing flies enter a unique, deep-sleep state, characterized by a rapid-onset, high arousal threshold, altered cycling characteristics, and rapid resumption of sleep following awakening.

### The Eye and Cryptochrome (Cry) Are Dispensable for Shaded Sleep Preference.

We next sought to understand the genetic basis for the shaded sleep preference at 25 °C. We reasoned that two classes of genes could be important for this behavior: those that comprise the circadian clock and those that are involved in light sensation. To test the latter, we first examined the shaded sleep preference of flies with major defects in visual transduction.

**Fig. 4.**
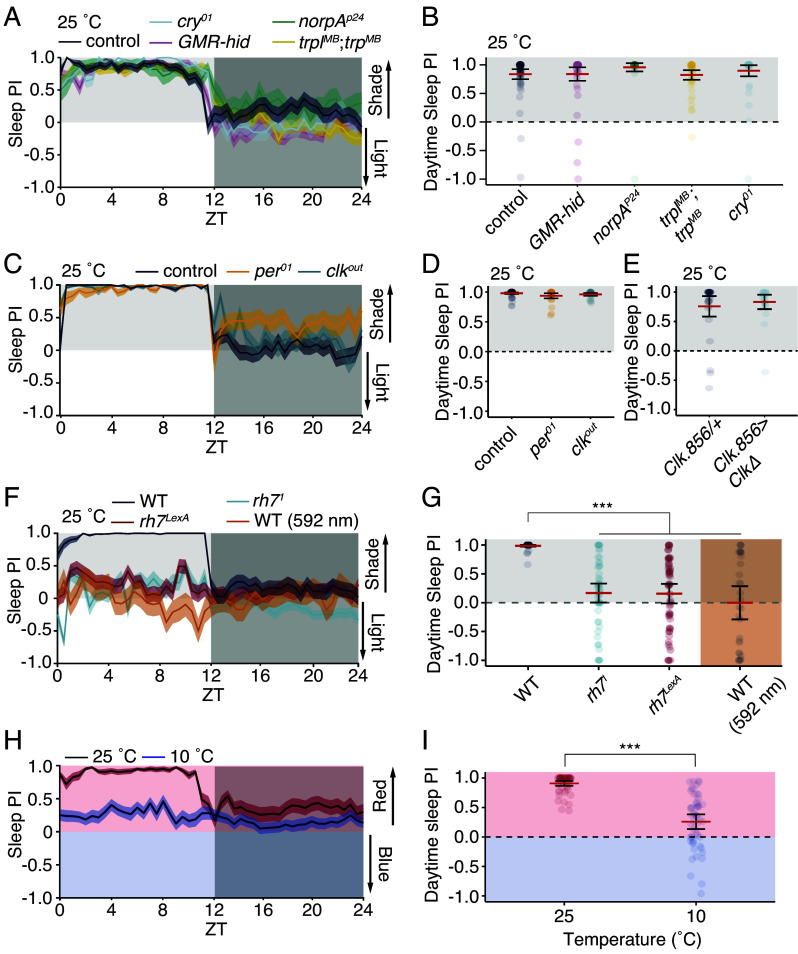
Rh7 is required for flies’ shaded sleep preference. Average sleep PIs ± SEMs (see *Materials and Methods* for formula) for the indicated genotypes. (*A*, *C*, *F*, and *H*) 24 h. (*B*, *D*, and *E*) Daytime sleep PIs for flies shown in *A*, *C*, *F*, and *H*, respectively. (*A* and *B*) n = 45 to 59 flies/genotype. (*C* and *D*) n = 27 to 58 flies/genotype. (*E*) Average sleep PI for flies expressing dominant negative CLK (CLKΔ) in circadian pace-maker neurons. n = 24 to 29 flies/genotype. (*F*) Sleep PI for *rh7* or wild-type flies housed under red (590 nm) light. (*G*) Daytime sleep PIs for flies shown in panel *F*. (*F* and *G*) n = 30 to 56 flies/genotype. In *A–G*, values > 0 indicate a preference for the shaded zone, and values < 0 for the unshaded zone. Data were analyzed first by aligned-rank transform two-way ANOVA (*P* < 0.001). Individual differences between groups were analyzed by aligned-rank transform contrast. (*H*) Red vs. blue sleep PI at 25 ° or 10 °C. (*I*) Daytime sleep PIs for H. Data were analyzed first by aligned-rank transform one-way ANOVA. n = 54 flies/temperature. *P* < 0.001.

The compound eye is composed of ~800 ommatidia, each of which houses eight photoreceptor cells. The phototransduction cascade is initiated by light activation of rhodopsin and subsequent activation of a phospholipase Cβ encoded by the *norpA* locus (38). The cascade then culminates with opening of two cation channels, TRP and TRPL (39–41). Surprisingly, flies harboring null mutations in *norpA* or double mutants for *trp* and *trpl* (*trpl^MB10553^*;*trp^MB03672^*) showed a daytime preference for shaded sleep at 25 °C that was not significantly different from the wild-type control (Fig. 4 *A* and *B*).

In addition to the compound eyes, flies possess three small eyes at the vertex of the head (ocelli) (42) and two Hofbauer–Buchner (H-B) eyelets (43). To test whether any eye structure is important for shaded sleep preference, we used flies that express a proapoptotic gene (*head involution defective*, *hid*) under the control of the *GMR* (*Glass Multimer Reporter*) promoter (44, 45), thereby eliminating the compound eyes, ocelli, and H-B eyelets. The shaded sleep preference of *GMR-hid* flies was similar to the control (Fig. 4 *A* and *B*), demonstrating that eye structures are not required for light aversion.

*Drosophila* can also detect light outside of the eye via Cry (46–48). Cry senses ultraviolet/blue light (450 nm peak) (49); however, flies with a null mutation in *cry* retained a strong preference for shaded sleep (Fig. 4 *A* and *B*).

### A Functional Circadian Clock Is Not Required for Shaded Sleep Preference.

To test whether the circadian clock impacts shaded sleep preference, we examined shade preference in clock mutants. The *Drosophila* circadian clock consists of at least two interlocked transcription–translation feedback loops executed by the transcription factors CLOCK and CYCLE, which heterodimerize to regulate the transcription of *period* (*per*) and *timeless* (*tim*) (50). In turn, PERIOD and TIMELESS heterodimerize and repress transcription of the *clock* and *cycle* genes. Disrupting any of these factors renders flies behaviorally arrhythmic when housed in conditions devoid of time-giving cues like light.

To test whether the molecular clock regulates the preference for shaded sleep, we tested flies with null mutations in either *per* (*per^01^*) or *Clk* (*Clk^OUT^*). Similar to the control, both mutants strongly preferred sleeping in the shade (Fig. 4 *C* and *D*; control PI = 0.98 ± 0.01; *per^01^*PI = 0.94 ± 0.02; *Clk^OUT^*PI = 0.96 ± 0.01). Similarly, disrupting the molecular clock in pacemaker neurons (the neurons in the brain that drive 24 h activity rhythms) by expressing a dominant negative CLOCK protein (51) failed to suppress the preference for sleeping in the shade (Fig. 4*E*). Thus, a functional circadian clock is not required for flies to prefer shaded sleep.

### Rhodopsin 7 (Rh7) Regulates Preference for Shaded Sleep.

Another extraocular light sensor is encoded by *rh7*, which is expressed in the brain (52) and in multidendritic neurons (53). This blue-light-sensitive opsin impacts circadian entrainment to blue light but does not signal through NORPA (52). As *norpA* mutants retained a strong preference for shaded sleep, we next tested whether Rh7 regulates this behavior using flies homozygous for either *rh7^1^* (52) or an allele that we created, *rh7^LexA^*, which contains *LexA* and *mini-white* positioned in frame following the endogenous initiation codon in the second exon of *rh7* (*SI Appendix*, Fig. S3*K*). Whereas the wild-type control showed a strong preference for shaded sleep (Fig. 4 *F* and *G*), both *rh7* mutants showed a minimal preference for sleeping in the shade at 25 °C (Fig. 4 *F* and *G*).

Rh7 is activated by blue light (52), so we tested flies housed in amber light (λ = 592 nm), which is outside of its spectral sensitivity. Unlike flies under a broad-spectrum white light, flies under amber light showed no preference for sleeping in the shade (Fig. 4*G*). Together, these results demonstrate that the preference for shaded sleep is specific for blue light, is independent of all eye structures and the circadian clock, and requires Rh7.

### Cool Temperature Suppresses *rh7-*Mediated Aversion to Blue Light.

To test whether shaded sleep preference is driven by avoidance of blue light, we placed a blue filter over one-half of the arena and a red filter over the other, such that flies could choose to sleep in a red or blue zone (*SI Appendix*, Fig. S4*A*). The red filter allowed peak emission of λ = 612 nm, with virtually no emission below λ = 575 nm (*SI Appendix*, Fig. S4*B*). The peak emission with the blue filter was 448 nm, although the spectrum had a smaller peak at λ = 510 nm, and narrow peak at λ = 612 nm (*SI Appendix*, Fig. S4*B*). Flies maintained at 25 °C strongly preferred sleeping in the red-shaded half of the arena (Fig. 4 *H* and *I*), reminiscent of flies choosing the shade rather than in white light. However, at 10 °C, flies showed only a minimal aversion to sleeping under blue light (Fig. 4 *H* and *I*). This suggests that the indifference to shaded sleep in the cold is the result of insensitivity to blue light.

**Fig. 5.**
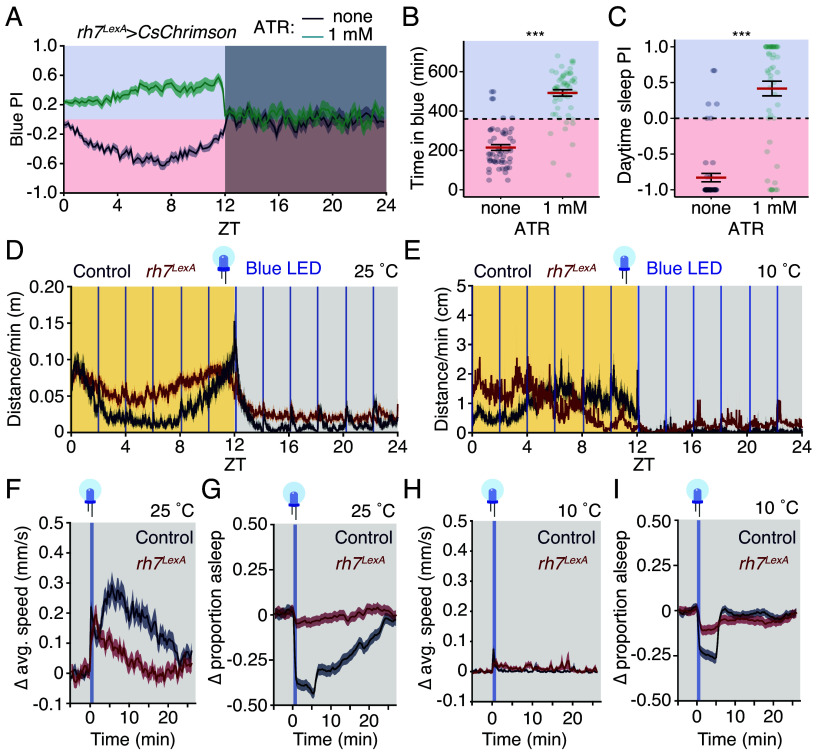
Cool temperature suppresses the wake-promoting effects of blue light. (*A*) Preference (average ± SEM) for the blue zone of *rh7* > csChrimson flies raised on food with (1 mM) or without (none) ATR (n = 54 to 57 flies per condition). (*B*) Time in the blue zone (average ± SEM) for flies in *A*. (*C*) Daytime PI for sleeping in the blue zone for flies in *A*. Data in *B* and *C* were analyzed using one-way aligned-rank transform ANOVA. (*D* and *E*) Activity profiles of control (blue lines) and *rh7^LexA^*(red lines) flies exposed to a blue-light pulse (vertical lines) every 2 h over one day at (*D*) 25 °C or (*E*) 10 °C. (*F*–*I*) Change in average speed (*F* and *H*) or proportion (*G* and *I*) of flies exposed to nighttime blue-light pulses at 25 °C (*F* and *G*) or 10 °C (*H* and *I*), normalized to prestimulus. (*F*–*I*) averages ± SEM of values following each late-night blue-light pulse (ZT 14 to 22), n = 50 to 59 flies/condition. ***P < 0.001.

To test whether the activation of *rh7-*positive neurons drives aversion to blue light, we used *rh7^LexA^* to express a transgene encoding a red-light-sensitive channelrhodopsin, CsChrimson (54), in *rh7*-positive neurons. We housed the flies in the same arena where they could choose between spending time in a red- or blue-shaded zone. However, we activated *rh7*-positive neurons on both halves of the arena. In the red-shaded zone, they were activated by CsChrimson, and in the blue-shaded zone by endogenous Rh7. Because CsChrimson requires the cofactor all-*trans* retinal (ATR) to be activated by red light, we compared the light preference of flies that had been raised on food with or without 1 mM ATR. Control flies (i.e., *rh7^LexA^* > *CsChrimson* flies raised on food without retinal) avoided spending time in blue light throughout the day (Fig. 5*A*), with only 215 ± 15 min, out of a possible 720 min spent outside of the red zone (Fig. 5*B*). In contrast, flies with functional CsChrimson avoided the red half of their enclosure, more than doubling the amount of time spent under blue light (Fig. 5*B*). Strikingly, we observed a similar trend for sleep preference. The control without ATR strongly preferred sleeping on the red side of their enclosure (Fig. 5*C*). In contrast, activating *rh7*-positive neurons with CsChrimson nearly reversed this preference, as these flies preferred to sleep under blue light (Fig. 5*C*). In total, these results reveal that the activation of *rh7*-positive neurons drives an aversive response.

### Cool Temperature Suppresses the Wake-Promoting Effect of Blue Light.

To test whether nondiapausing flies avoid sleeping under blue light because it promotes wakefulness, we housed flies in 12L:12D cycles, with daytime light from a 592 nm light source, which is outside of the spectral sensitivity of Rh7. Every 2 h, we delivered five 3-s pulses of blue light and measured the behavioral responsiveness to these stimuli.

At 25 °C, control flies responded to nighttime blue-light pulses with an immediate startle response (i.e., an increase in activity coincident with the start of the light pulses) followed by elevated activity that took ~25 min to return to baseline (Fig. 5 *D* and *F*). There was a commensurate 45% reduction in sleep immediately following the light pulse (Fig. 5*G*). The *rh7* mutant flies also exhibited a startle response to blue light; however, their activity levels rapidly returned to baseline (Fig. 5*F*), suggesting that *rh7* is required for the prolonged stimulatory effects of blue light. Blue-light pulses failed to change the proportion of *rh7* mutant flies that were asleep, indicating that Rh7 is required for the wake-promoting effects of blue light (Fig. 5*G*).

Unlike at 25 °C, control flies at 10 °C were relatively indifferent to nighttime blue light (Fig. 5*E*). Although there was a minor startle effect (a small, transient increase in average walking speed), the movement of these flies rapidly returned to baseline (Fig. 5 *E* and *H*). Similarly, blue light did not substantially reduce sleep in flies at 10 °C, which rapidly resumed sleep after being roused by the light stimulus (Fig. 5*I*). At 10 °C, mutating *rh7* had little effect (Fig. 5 *H* and *I*). Together, these results reveal that a cool temperature can overcome the wake-promoting effects of blue light, which likely contributes to the relative indifference of flies at 10 °C to sleeping in the shade.

### Diapause Conditions Induce Neuronal Markers of High Sleep Pressure.

Like in mammals, sleep in *Drosophila* can be described by a two-process model consisting of the internal circadian clock, which affects the timing of sleep throughout the day, and the sleep homeostat, which imparts sleep pressure as a consequence of the previous length of wakefulness (55). Notably, flies experiencing high levels of sleep pressure, such as those that have been sleep-deprived for a night, respond by increasing their total levels of sleep, and by experiencing deeper sleep than normal (56, 57). Because we observed that flies at a diapause-permissive temperature exhibit behavioral characteristics of a state of high sleep pressure (need) including an increase in arousal threshold and total sleep, as well as a decrease in sleep latency, we wondered whether they display increased expression of neuronal markers associated with sleep drive.

**Fig. 6.**
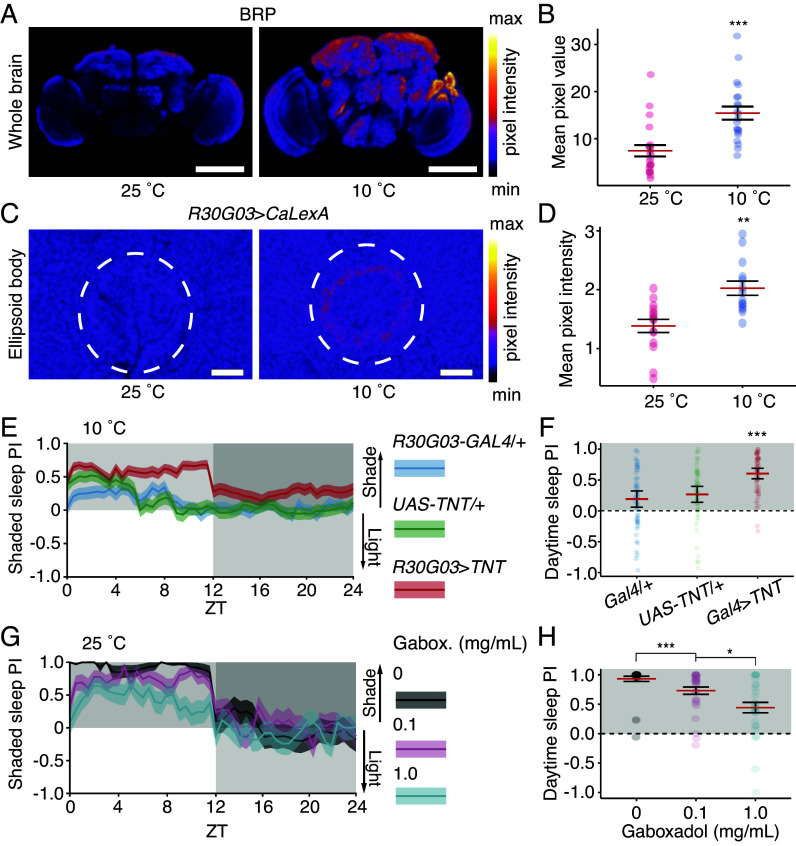
Diapause actively imparts sleep pressure, which is sufficient to overcome aversion to light. (*A*) Representative BRP staining in the wild-type at 25 °C or 10 °C. (Scale bar, 100 µm.) (*B*) Quantification of BRP staining. Data were compared via the Wilcoxon rank sum test. n = 21 brains/temperature. (*C*) Representative images of *R30G03-Gal4*/+ > *CaLexA*/+ from flies at 25 °C or 10 °C. (Scale bar, 10 µm.) (*D*) Quantification of pixel intensity from *R30G03-Gal4*/+ > *CaLexA*/+ flies maintained at 25 °C or 10 °C. Wilcoxon rank sum test. n = 14 to 15 brains/temperature. (*E*) Sleep PI of R30G03 > *TNT* flies at 10 °C. (*F*) Daytime sleep PI of R30G03 >*TNT* flies at 10 °C. (*E* and *F*) n = 52 to 56 flies/genotype. (*G*) Sleep PI of wild-type flies given gaboxadol. (*H*) Quantification of daytime sleep PI of wild-type flies given gaboxadol. (*G* and *H*) n = 29 to 30 flies/genotype. ***P < 0.001. (*D*, *F*, and *H*) Data compared via aligned-rank transform ANOVA with group differences compared via aligned-rank transform contrasts with Bonferroni multiple testing correction.

To assess expression of sleep pressure markers, we examined Bruchpilot (BRP), a presynaptic active-zone protein that drives sleep pressure and depth (58). We housed flies at either 25 °C or 10 °C for ~36 h and at ZT 4 we stained dissected brains with anti-BRP. In wild-type flies at 10 °C, BRP expression was elevated twofold relative to flies at 25 °C (Fig. 6 *A* and *B*). Similarly, BRP expression was increased in *rh7* mutant flies in response to cold (*SI Appendix*, Fig. S4 *C–E*). This demonstrates that the deep sleep-like behavior observed in diapause conditions is also accompanied by a neuronal marker of high sleep pressure. Furthermore, these results show that *rh7* mutants accumulate sleep pressure in a manner that is similar to wild-type flies in diapause conditions.

Because BRP is reported to drive sleep pressure in part by activating R5 neurons in the ellipsoid body (59), we wondered whether flies in diapausing conditions showed elevated R5 neuron activity. To address this question, we used a genetically encoded Ca^2+^ reporter, CaLexA, which consists of the LexA transcription factor fused to the Ca^2+^-responsive element Nuclear Factor of Activated T cells (NFAT) (60). Sustained elevation of Ca^2+^ causes LexA::NFAT to translocate into the nucleus and drive expression of *LexAop-GFP* (Green Fluorescent Protein), allowing *GFP* expression to serve as an indirect marker of neuronal activity. We observed that flies maintained in diapausing conditions for ~36 h showed elevated levels of GFP in R5 cells, suggesting that 10 °C increases the activity of these neurons (Fig. 6 *C* and *D*). Notably, the activity of R5 neurons within the ellipsoid body plays a crucial role in generating sleep drive within the fly (61), indicating that the state is actively maintained rather than a passive effect of cool temperature and providing a potential mechanism for the diapause deep-sleep state.

### Sleep Pressure Is Sufficient to Overcome Flies’ Aversion to Light.

Because flies in diapausing conditions exhibit both behavioral and neuronal markers of high sleep pressure, we wondered whether an elevated level of sleep pressure would be sufficient to suppress the preference for shaded sleep. To address this idea, we first expressed tetanus toxin (*UAS-TNT*) under the control of the R5 neuron driver *R30G03*-*Gal4*, which inhibits neurotransmitter release in these cells and is reported to partially alleviate sleep pressure (61). At 10 °C, *R30G03*> *TNT* flies slept significantly less per day compared to the controls (*SI Appendix,* Fig. S4 *F* and *G*) and had an elevated preference for shaded daytime sleep (Fig. 6 *E* and *F*).

To determine whether increasing sleep pressure through a mechanism independent of low temperature would overcome the aversion to sleeping in the light, we added a GABA_A_ receptor agonist, gaboxadol, to the food of flies maintained at 25 °C (62). The gaboxadol increased total sleep in a dose-dependent fashion. In the absence of gaboxadol, the flies slept 252 ± 11 min during the daytime (*SI Appendix*, Fig. S4 *H* and *I*). However, flies fed gaboxadol significantly increased their sleep, especially during the day (*SI Appendix*, Fig. S4 *H* and *I*). Accompanying this increase was a decrease in their preference for shaded sleep. Wild-type flies on food without gaboxadol displayed a strong preference for shaded sleep (Fig. 6 *G* and *H*), whereas flies fed gaboxadol had a dose-dependent reduction in their PI (Fig. 6 *G* and *H*). Alleviating sleep pressure by blocking R5 neurons increased shaded sleep at 10 °C, and enhancing sleep pressure with gaboxadol dissipated this preference at 25 °C; preference for sleeping in shade can be modulated by sleep drive.

Notably, gaboxadol treatment did not reshape the sleep pattern such that it mimicked flies at 10 °C (*SI Appendix*, Fig. S4*H* vs. *SI Appendix,* Fig. S2*H*; 10 °C). Gaboxadol-treated flies exhibited slightly increased sleep during the middle of the day (*SI Appendix*, Fig. S4*H*), whereas flies in diapause were most active during this time (Fig. 1*B*). This indicates that the diapause activity/sleep rhythm is not solely based on increased sleep pressure, further highlighting the multifarious effects of cool temperature.

## Discussion

The results reported here support the emerging concept that *Drosophila* diapause is a comprehensive program that is initiated by neuronal sensing of environmental temperature and results in coordinated changes in behavior, metabolism, reproduction, and lifespan. Here, we report that the same range of low temperatures (10 to 15 °C) that induces reproductive arrest and extends lifespan in various *Drosophila* species has a strong effect on circadian behavior and sleep, independent of JH. Cool temperatures do not trivially reduce all neuronal activity or simply immobilize the animals. Rather, 10 to 15 °C actively increases sleep pressure, causing flies to rapidly fall into a deep sleep from which it is difficult to rouse. Nevertheless, they maintain rhythmic behavior under light–dark cycles, though dramatically altered from nondiapausing flies. Their sleep preferences change too, as they no longer prefer sleeping in the shade over a well-lit environment in the afternoon. Furthermore, their sleep is so deep that their arousal threshold does not seem to decrease no matter how long they have been asleep. These features are somewhat reminiscent of hibernating mammals, which show extended bouts of non-REM-like sleep with intermittent periods of wakefulness (3).

### Basis for the Profoundly Altered Daytime Activity Pattern of Diapausing Flies.

The circadian activity pattern of diapausing flies is strikingly different from nondiapausing flies. It is well established that nondiapausing flies have peak activities near dawn and dusk with a midday siesta, and this crepuscular behavior is under circadian control (21, 50). However, the activity pattern of diapausing flies is opposite with peak activity during the midday. Thus, diapause-inducing conditions profoundly shift the circadian activity pattern while maintaining rhythmicity. Furthermore, we found that temperatures ≤15 °C, the same temperatures that arrest egg production (17), caused the shift in circadian rhythm. Two different classes of circadian neurons have been reported to alter their activity at 10° (17, 18), suggesting a mechanism by which cool temperatures might alter circadian activity. We found that, upon shifting flies from 25 to 10 °C, the total level of activity changed abruptly, whereas completely shifting the evening activity peak required a full circadian cycle at the cooler temperature. It will be interesting in the future to examine the effect of cool temperatures on transcription, translation, and protein stability of core clock components to further elucidate the mechanism of the temperature-induced shift in circadian activity.

The midday activity in diapausing flies appears to be due to advancement of the evening activity peak, accompanied by a virtual elimination of the morning activity. Consistent with this proposal, the single activity peak is not precisely in the middle of the day but shifted slightly toward dusk. This was most apparent when we maintained diapausing flies under 16L:8D cycles. Under these extended day cycles, there was partial overlap of the activity peak with the evening peak for nondiapausing flies, but no overlap with the morning peak.

In the case of nondiapausing flies, the siesta is thought to reduce the risk of dehydration during the hottest part of the day. However, cool temperatures reduce that risk, offering a plausible selective advantage for increased activity during the middle of the day at 10 °C.

### Sleeping in the Shade Depends on an Extraocular Role for Rh7.

We uncovered a daytime behavior that we suggest might also minimize dehydration at 25 °C. Using an assay that we developed, we found that nondiapausing flies have a strong preference to sleep in the shade, and this bias is reduced in diapausing flies in the morning and virtually eliminated after noon. The second half of the day, when flies in diapause are indifferent to sleeping under blue light, coincides with their deepest sleep. Somewhat surprisingly though, the afternoon/evening is when flies in diapause are also most active. This indicates that during this time, flies have short but deep sleep bouts, whereas during the first half of the day flies in diapause have longer but shallower sleep.

There are at least two non-mutually-exclusive possibilities that could explain why nondiapausing flies have such a substantial preference to sleep in the shade. The first is that sleeping in a lighted environment is aversive. The second possibility is that light promotes wakefulness, and flies are unable to fall asleep when exposed to it. Our data support the model that flies prefer to sleep in the shade because blue light stimulation of *rh7* neurons causes both aversive behavior and arousal. Our results are consistent with a previous finding that *rh7* mutants are indifferent to blue light during the day (53), although this study tracked the absolute location of the flies over the span of a day and did not distinguish whether a fly was asleep. Here, we demonstrate that daytime blue-light avoidance is primarily driven by a preference for where to rest, and this behavior depends on Rh7.

The question arises as to why diapausing flies lack the Rh7-mediated preference for daytime shaded sleep. A plausible explanation is that increased sleep pressure overwhelms their aversion to sleeping under blue light. Flies in diapause display increased activity of R5 neurons and expression of BRP, both of which indicate increased sleep drive. Partially alleviating sleep drive, by blocking the activity of R5 neurons, increased the preference for shaded sleep at 10 °C. Conversely, pharmacologically increasing sleep drive in flies at 25 °C decreased this preference. These data support the model that the effects of cool temperature on sleep in diapausing flies are active responses. We propose that at diapause-inducing temperatures flies are relatively indifferent to sleeping in shade owing to elevated sleep pressure, rather than an inability to sense blue light.

### Cool Temperatures Induce Deep Sleep.

While sleep has been extensively studied in *Drosophila* under optimal growth conditions, understanding the impact of adverse environments on fly sleep is a frontier (21). Our work reveals that flies maintained at diapause-inducing temperatures enter a far deeper sleep state than nondiapausing flies, as their sleep is characterized by a much higher arousal threshold, which is one of the defining characteristics of a deep-sleep state (63). Moreover, this deep-sleep state occurs during both the day and night. This finding is reminiscent of hibernating animals, which enter a deeper state of rest than when they are not hibernating (64).

Additionally, the increase in the arousal threshold in diapausing flies is profound even after short periods of inactivity. Remarkably, after just 15 to 60 s of inactivity, diapausing flies exhibit an arousal threshold that is greater than the highest average threshold ever exhibited by nondiapausing flies. Moreover, the sleep latency of diapausing flies remains low at all times during the day and night whereas in nondiapausing flies, in which the sleep latency is short only at night, and for a brief period during their siesta. Even during these periods, the sleep latency of nondiapausing flies is not as short as in diapausing flies. Thus, diapausing flies enter a persistent deep-sleep state that is not observed in nondiapausing flies.

### Implications for Understanding Diapause.

Classically studied diapause traits include slow growth and development, reproductive arrest, increased stress resistance, and lifespan extension (2, 8, 12). Less studied are the effects of diapause-inducing conditions on behavior. At first glance, it seems surprising that temperature, rather than photoperiod, alters the circadian rhythm so profoundly in diapause-inducing conditions. Yet, reproductive dormancy in *Drosophila melanogaster* is also known to depend more strongly on cool temperatures than on short day length (13, 17, 18). Some investigators define diapause as a photoperiod-dependent reproductive arrest and prefer to call temperature-dependent effects “dormancy.” Temperature may be the more salient information because temperature rather than photoperiod directly affects growth rates and reproductive success.

The influence of photoperiod seems to be selected for at high latitudes (9). Both low and high latitude strains of *Drosophila montana* arrest reproduction at low temperatures, whereas only high latitude strains show a strong dependence on photoperiod. An advantage of responding to photoperiod rather than directly to environmental temperature is that animals can anticipate and prepare in advance for harsh conditions using photoperiod, which is less susceptible to unseasonal variation. However, a potential disadvantage to relying on photoperiod is that during periods of climate change, photoperiod could become a poor predictor of temperature, which is the feature with a direct impact on survival.

Cool temperatures affect multiple aspects of fly behavior, physiology, reproduction, and lifespan, and outlines of the cellular and molecular mechanisms are beginning to emerge. Cool temperatures decrease the activity of multiple circadian neurons, which in turn affect production of hormones such as insulin-like peptides and JH, which is a key regulator of vitellogenesis (17, 18). The effects of cool temperatures on sleep and activity reported here are independent of the hormone JH (1, 8). We previously found that cool temperatures also arrest germline stem cell division independently of JH (13). Together, the results lead to a model in which cool temperatures are initially sensed by circadian neurons, exerting multiple effects on fly physiology, behavior, and ovarian development that together represent a holistic, adaptive response.

## Materials and Methods

### *Drosophila* Stocks and Maintenance.

Flies were maintained under standard conditions. Flies were allowed to acclimatize to their arena for ≥16 h prior to behavioral assays. Additional details are found in *SI Appendix, Materials and Methods*.

### DAM Assay.

To record circadian activity profiles, 5- to 10-d-old mated female Canton S flies were recorded using the DAM system (Trikinetics) (19). Additional information for DAM assays can be found in *SI Appendix*.

### Behavioral Arena for Activity/Sleep Monitoring and Shade Preference.

We designed a custom behavioral arena to track movements, which also served to assess preference for shade, red vs. blue light, and startle responses to vibration or light. The stereolithography (also known as STL) file for the behavioral arena can be found at the following URL: https://github.com/Craig-Montell-Lab/Meyerhof-et-al.-2024-/tree/main/SunSeekerPrintFile.

Additional information regarding this assay, as well as our video tracking approach, can be found in *SI Appendix*.

### Vibration Sleep Arousal Threshold.

To test the sleep depth at 25 °C and 10 °C, we housed flies in the behavioral arena and subjected them to five gradually increasing vibration stimuli by applying 1 to 5 V, via pulse-width modulation from an Arduino Uno (Elgoo) microcontroller, to a Mosfet transistor (WeiMeet) that controlled four vibrating motors wired in parallel and placed at each corner of the arena (*SI Appendix*, Fig. S3*A*). We used a triple-axis accelerometer (ADXL326; Elgoo) wired to an Arduino Uno (Elgoo) to measure the *g* force on the arena motors (*SI Appendix*, Fig. S3 *C* and *D*).

### Blue Light Sleep Arousal.

To test the wake-promoting effects of blue light (Fig. 5 *D*–*I*), we subjected flies to five 3 s light pulses with a 10 s interpulse delay, once every 2 h. We used a blue (445 nm) LED light strip (85 lx; American Bright Optoelectronics Corp.) controlled via an Arduino Uno (Elgoo), which received commands from a custom Matlab script (https://github.com/Craig-Montell-Lab/Meyerhof-et-al.-2024-/tree/main/SunSeeker_LightPulse) that was integrated into our tracking program.

### Immunohistochemistry and Confocal Microscopy.

We immuno-stained adult brain whole mounts. Primary antibodies: anti-GFP (chicken, Invitrogen, A10262, 1:1,000 for CaLexA expression comparison), anti-nc82 (mouse, Developmental Studies Hybridoma bank, nc82 concentrate (1:50 for BRP expression comparison, otherwise 1:250) Secondary antibodies: Alexa Fluor 488 conjugated goat anti-chicken (1:1,000, Invitrogen, A11039), Alexa Fluor 633 conjugated goat anti-mouse (1:1,000, Invitrogen, A21050). Images were acquired using a Zeiss LSM 900 confocal at 20× magnification (PApo 20×/0.8 objective). The complete protocol for immunostaining and confocal image acquisition can be found in *SI Appendix, Immunohistochemistry and Confocal Microscopy*.

### Optogenetics.

*rh7^LexA^* virgin females were crossed to *LexAop-CsChrimson* males, which were raised on standard fly food with or without the addition of 1 mM ATR (Sigma; R2500). After 4 d, the parents were removed from the fly vial and the progeny were shifted to constant darkness to complete development. After eclosion and mating, female *rh7 > CsChrimson* flies were transferred to our behavioral arena which contained a red- and blue-light filter (Neewer.com).

### Testing Effect of Gaboxadol on Shaded Sleep Preference.

To test the effect of sleep pressure on shaded sleep preference, we loaded flies into our behavioral arena with a sucrose food source (5% sucrose + 1.5% agar) that contained either 0.1 or 1 mg/mL of gaboxadol (Cayman; 16355). Mated female Canton S flies were aspirated via a mouth pipette into the arena at ZT 8 the day prior to the start of the recording, where they had ad libitum access to gaboxadol-laced food throughout the experiment.

### Quantification and Statistical Analysis.

Sample sizes and statistical tests are provided in the figure legends. “n” denotes the number of flies examined. All statistical tests were performed in R (version 4.1.0). Based on our experience and common practices in this field, we used a sample size of n ≥ 20 flies for circadian locomotor and sleep analyses. Nonparametric aligned-rank ANOVA was performed using the “ARTool” library. Parametric ANOVA was performed using the “stats” R package. Plotting was performed using either the “ggplot2” R library or Matlab 2021a. Error bars display SEMs unless otherwise indicated in the figure legend. We set the significance level, α = 0.05. Asterisks indicate statistical significance: *P < 0.05, **P < 0.01, and ***P < 0.001.

## Supplementary Material

Appendix 01 (PDF)

Movie S1. Example video displaying video tracking of Canton S. flies at 25 °C under white light at ZT 4 in the shade preference arena.

## Data Availability

All study data are included in the supporting information and source data have been uploaded to a public repository at doi:10.5061/dryad.0p2ngf28n (65).
