# Supplementary material for: Altered circadian rhythm, sleep, and rhodopsin 7–dependent shade preference during diapause in Drosophila melanogaster
Source: Proc Natl Acad Sci U S A. 2024 Jun 25;121(27):e2400964121. doi: 10.1073/pnas.2400964121 (PMC11228485; doi:10.1073/pnas.2400964121)
Supplement: Supplementary file 1 — Appendix 01 (PDF) [file pnas.2400964121.sapp.pdf]

PNAS

Supporting Information for

Altered circadian rhythm, sleep, and *rhodopsin 7*-dependent shade preference during diapause in *Drosophila melanogaster*

Geoff T. Meyerhof, Sreesankar Easwaran, Angela E. Bontempo,
Craig Montell and Denise J. Montell

Denise J. Montell
Email: denise.montell@lifesci.ucsb.edu

This PDF file includes:

SI Materials and Methods
Figures S1 to S4
Legend for Movie S1
SI Reference

SI Materials and Methods

***Drosophila* stocks and husbandry.** Stocks and crosses were maintained in temperature (25 °C) and humidity (60%) controlled incubators (Darwin Chambers) under a 12 hr light (~750 lux):12 hr dark cycles on standard fly food (molasses, yeast, and cornmeal). In this paper, the following stocks were used: The following fly stocks were used: Canton S (BDSC#64349); w^{1118} (BDSC#5905); $trpl^{MB10553}$;$trp^{MB03672}$ (C. Montell lab); per^{01},w^{1118} (BDSC#80917); w^{1118},$norpA^{P24}$ (C. Montell lab); w^{*};clk^{out} (BDSC#56754); *UAS-Clk*∆ (BDSC#36319); *R30G03-Gal4* (BDSC#49646); *CaLexA* (*lexAOP*-GFP;;*UAS-LexA::VP16::NFAT*/TM6B) (BDSC#66542); *clk.856-Gal4* (BDSC#93198); *UAS-TNT* (BDSC#28838); *GMR-hid* (C. Montell lab); *lexAOP-CsChrimson* (BDSC#55137).

Generation of $rh7^{LexA}$ flies. To generate the $rh7^{LexA}$ allele we used CRISPR/Cas9 to insert the *LexA* gene in frame in place of the first 179 base pairs (bp) of the second exon, following the endogenous translation start site. The construct also included the *mini white* (w^+) gene. The donor plasmid was pBPLexA::p65Uw (Addgene plasmid 26231). The two homology arms were amplified from genomic DNA prepared from w^{1118} using the following primers:
forward primer for 5′ arm: GAAAAGTGCCACCTGCTCACATGCGAAGGGGGAAT; reverse primer for 5′ arm: CATTTTGATTGCTAGGTGGTACTTGGCCAAATATTTACGAGC;
forward primer for 3′ arm: GACAAGCCGAACATAGAGAATCCCACAAATGAATTTCTGCAAG,
reverse primer for 3′ arm: CTAGGCGCGCCCATAGTGAATGTAGGCAAGGACCACG.
The 5′ arm was inserted between the AatII and NheI sites, and the 3' arm was inserted into the NdeI site. The 5' arm included a modified Kozak sequence: ACCAC. The guide-RNA targeting sequence was: GATGGCCTCCATGACTGACT.

***Drosophila* Activity Monitor assay.** For DAM assay experiments, flies were individually loaded into clear glass tubes with a sucrose food source (5% sucrose + 1.5% agar) on one end and a cotton plug on the other. Flies were allowed to acclimatize for >24 hours prior to the start of a 4-day-long recording. Their activity was logged on a computer using the *Drosophila* Activity Monitor system software and plotted in 5- or 30-minute bins using the Rethomics R software package (1). Moving/30 or 5 minutes represents the proportion of time in which there were beam breaks per 30 minutes (or 5 minutes). In these experiments, flies were housed under white LED lights (Minger #H6150) at ~1700 lux or up to 400 lux, as indicated. Dead or sick flies (i.e. flies with <14 beam breaks/day) were excluded from the analysis.

In order to test the effect of ramping light intensity on Drosophila activity in diapause, we designed a custom LED light controller that was synchronized to the clock on the computer that controlled the DAM assay. Specifically, a Matlab (2021a) program controlled a pulse-width modulation pin on an Arduino Uno (Elgoo). This allowed for various voltages to be applied to the gate of a mosfet transistor wired to LED lights, and, subsequently, change the intensity of the lights. To approximate a more naturalistic change in illuminance throughout the day, we programmed the light intensity to follow a half sine wave (0 to $\boldsymbol{\pi}$) from ZT 0–12. In this paradigm, the light intensity gradually increased from ZT 0–6 and gradually decreased from ZT 6–12. From ZT 12–24, the flies received no light, mimicking nighttime.

Behavioral arena for activity/sleep monitoring and shade preference. Our custom behavioral arena for monitoring activity and sleep was 102 mm x 119 mm x ~7 mm and housed 30 flies, each in a rectangular enclosure (44 mm x 6 mm x 6 mm) with ad libitum access to a 5% sucrose in 1.5% agar. The face of the arena contained grooves to fit a clear acrylic sheet (McMaster #8560K171) that prevented the flies from escaping. Flies were anesthetized with CO_2 in order to load them into their enclosure, and then allowed ~24 hours to recover prior to the start of the recording. For experiments testing flies' preference for shade, we covered one half of the arena with a 0.9 neutral density filter, which blocked visible light but was nearly transparent to our IR light source. We used a similar approach in experiments testing the preference for red vs. blue, where one half of the arena was covered with one of the light filters. In order to visualize flies at

night, as well through a light filter, the arena was backlit with an 850 nm near-IR LED light (Waveform #7031) and recorded with an ELP 2MP webcam affixed with an IR-pass filter (Heliopan 850 nm). A CCS100 Spectrometer (Thorlabs) was used to measure the relative light intensity in our various experimental conditions (*SI Appendix*, Fig. S1*B* and Fig. S4*A*).
To analyze movement and sleep in flies in our arena, we wrote custom Matlab scripts (https://github.com/Craig-Montell-Lab/Meyerhof-et-al.-2024-). Dead or sick flies were censored from the analysis and were defined as any fly with a bout of immobility that lasted >6 hours. Data are displayed in 30-minute bins unless otherwise indicated, and the preference index for shaded sleep was defined as:
$Sleep\ PI = (sleep\ in\ shade\ - sleep\ in\ light)/total\ sleep.$
$Shade\ PI = time\ in\ shade - time\ in\ light/ten\ minute\ period.$

Video tracking. To track the position of flies in the aforementioned arena we wrote custom scripts in Matlab 2021a (Mathworks), which is available at https://github.com/Craig-Montell-Lab/Meyerhof-et-al.-2024-. Flies were recorded at ~2 frames per second in grayscale at a resolution of 1080 x 19020 from a webcam placed 45 cm away from the arena. Fly positions were recorded in real time via a movement-based tracking algorithm. To identify foreground pixels (i.e., pixels corresponding to moving flies) over multi-day experiments, we implemented a dynamic background model. The background model was created by taking the average pixel intensity at each pixel location from 30 consecutive frames. Then, a new frame was read in and the absolute difference was taken between it and the background model. Foreground pixels were defined as those greater than an empirically determined threshold (6 pixels):

$$foreground\ pixels = |background\ model - new\ frame| > threshold$$

Following this step, the oldest frame in the background model was replaced by the new frame and the process repeated. To reduce the chances of misassigning background pixels as foreground, we only considered contiguous foreground pixel regions (blobs) >10 pixels^2 and <500 pixels^2 that occurred within a fly enclosure. An example of the tracking is given in Supplementary Video 1.

Immunohistochemistry and confocal microscopy. To prepare samples for imaging, whole flies were fixed in 4% paraformaldehyde (Electron Microscopy Sciences) and 0.3% Triton X-1000 in 1x PBS for 1 hour at 4 °C. Samples were washed 2 times in 0.3% PBST (0.3% Triton X-1000 in 1x PBS). Then the brains were dissected into fresh, chilled PBST, blocked in 0.3% PBST containing 5% normal goat serum (NGS) for 1 hour at room temperature, and incubated with primary antibodies in 0.3% PBST + 5% NGS for 24 hours at 4 °C. Samples were then subjected to three 30-minute washes, and incubated in secondary antibodies in 0.3% PBST + 5% NGS overnight (~16 hours) at 4°C. After three additional 30 minute washes, the brains were mounted on glass slides with VECTASHIELD anti-fade mounting media (Vector Labs, catalog. H-1200).

Images were acquired on a Zeiss LSM 900 confocal microscope at 20x magnification at a resolution of 3064 x 3064 pixels. Within each experiment, images were acquired using identical laser power, gain, Z-stack depth (1µm), and scan speed using the CO-2Y setting (Zen Blue). Unprocessed images were exported from Zen Blue as .czi files and imported to FIJI (ImageJ). To measure signal intensity, the background signal of each optical slice in a stack was subtracted (except in the case of BRP experiments), a maximum intensity projection was generated, and the mean pixel intensity of each outlined ROI (whole brain or EB) was calculated.

Methoprene treatment. We treated flies with the JH analog methoprene and monitored their ovary development during diapause compared to the vehicle treatment control. Initially, we collected virgin CS females (10 flies per vial; less than 6 hours old) from flies reared in optimal conditions (12L:12D; 25°C) and transferred them to diapause conditions (8L:16D; 10°C). Immediately after moving, each vial was provided with a cotton swab saturated with either 10 µl of 20 µg/µl methoprene pestanal (from Sigma #33375) in ethanol or 10 µl of ethanol for the vehicle control. The cotton swab was replenished with methoprene at the same concentration every other day. After 10 days, each group of flies was dissected, and ovaries were imaged using a Zeiss Axio Zoom microscope. The ovary size was then compared by measuring the area of ovaries using Fiji software.

Figure S1

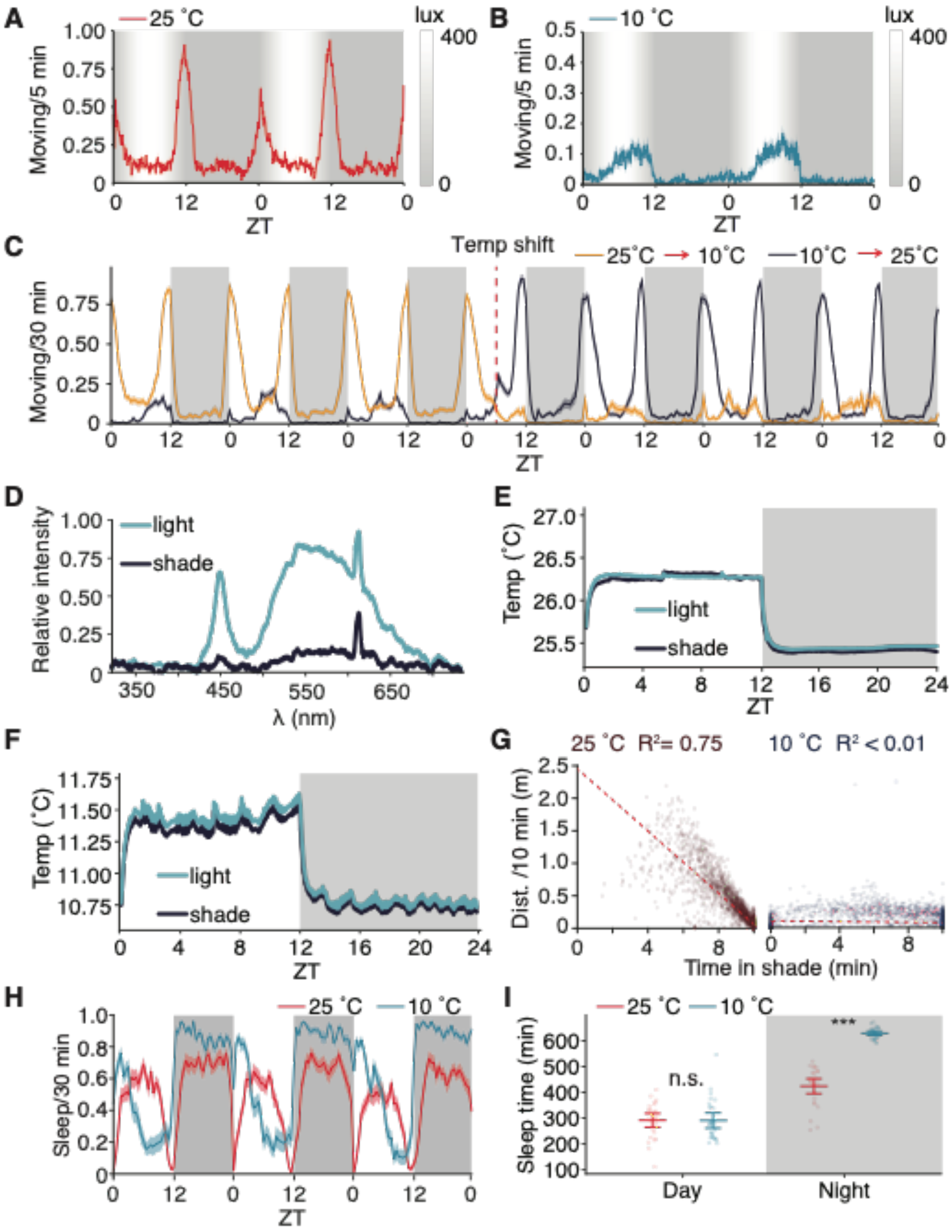

A
25 ˚C
Moving/5 min
lux
400
0
ZT
B
10 ˚C
C
Temp shift
25˚C → 10˚C
10˚C → 25˚C
Moving/30 min
D
light
shade
Relative intensity
λ (nm)
E
Temp (˚C)
F
G
25 ˚C R²= 0.75
10 ˚C R² < 0.01
Dist. /10 min (m)
Time in shade (min)
H
Sleep/30 min
I
Sleep time (min)
n.s.
***
Day
Night

Fig. S1. Ramping light, temperature shift, light intensity, distance vs. shade correlation, and sleep of flies in diapause. Circadian activity of flies at a warm or cool temperature when housed under a ramping light that gradually increases (ZT 0–6) and decreases (ZT 6–12). *(A* and *B)* Circadian activity of flies under a ramping light at 25℃ (*A*) and 10℃ (*B*). Light intensity gradually increased to 400 lux from ZT 0–6 and gradually decreased to 0 lux from ZT 6–12 (see Methods for additional details). (*C)* Effect of rapid temperature shift on flies' activity rhythms. Red dashed line signifies a shift in temperature from either 25℃ to 10℃, or 10℃ to 25℃. (*D*) Relative light intensity of shaded and unshaded zone in arena. (*E* and *F*) Temperature recording from behavioral arena (Fig. 2*A*) from shaded and unshaded zones over the span of a day when the arena was housed in an incubator set to 25℃ (*E*) or 10℃ (*F*). (*G*) Correlation between distance/10 minutes and time spent in the shade/10 minutes from flies at 25 °C (left) or 10℃ (right). Each dot represents a 10 minute bin from the daytime in a three-day long recording. Red dashed line represents the linear best fit line. (*H*) Three- day sleep profile of flies housed at 25℃ (red) or 10℃ (blue). Shading represents SEM. (*I*) Quantification of average sleep time during three-day long recording, separated by day and night. Error bars indicate the 95% confidence interval around the mean. Data were analyzed first by Aligned Rank Transform Two-Way ANOVA, examining factors of time, temperature, and time:temperature interaction. Individual differences between groups were analyzed by Aligned Rank Transform Contrast, with Bonferroni multiple testing correction. *A* and C*:* n=48–55 flies/condition. *C*: n=43–59 flies/condition. *H–I*: n = 27–29 flies/temperature. ****P*<0.001.

Figure S2

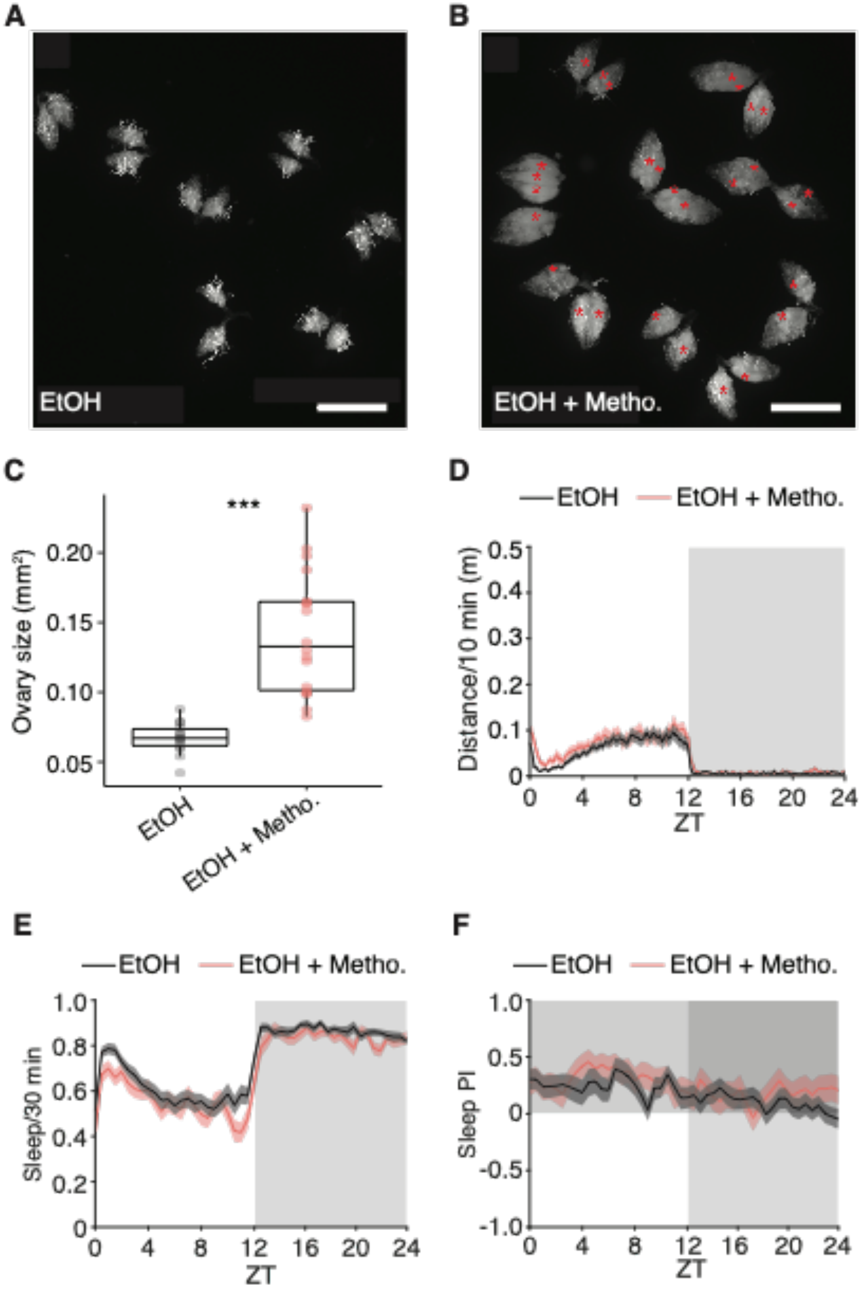

A
B
EtOH
EtOH + Metho.
C
***
Ovary size (mm²)
0.20
0.15
0.10
0.05
EtOH
EtOH + Metho.
D
EtOH
EtOH + Metho.
Distance/10 min (m)
0.5
0.4
0.3
0.2
0.1
0
0 4 8 12 16 20 24
ZT
E
EtOH
EtOH + Metho.
Sleep/30 min
1.0
0.8
0.6
0.4
0.2
0
0 4 8 12 16 20 24
ZT
F
EtOH
EtOH + Metho.
Sleep PI
1.0
0.5
0
-0.5
-1.0
0 4 8 12 16 20 24
ZT

Fig. S2. Methoprene treatment reverses ovarian arrest in diapause but fails to modify circadian behaviors. (*A*) Darkfield micrograph of the whole ovary from flies treated with ethanol (drug vehicle control). Scale bar is 1 mm. (*B*) Darkfield micrograph of whole ovaries from flies treated with ethanol and 100 µg of methoprene. Scale bar is 1 mm. Asterisks label egg chambers ≥ stage 12. (*C*) Quantification of ovary size as area (mm^2), performed using Fiji software. Box plot represents median, 25th percentile, and 75th percentile, with whiskers extending to either to the min/max value or 1.5 x the interquartile range. n=14–18 ovaries per condition. Groups compared via Wilcoxon rank sum test. ****P<0.001.* (*D*) Circadian activity profile of vehicle-treated (black) or methoprene-treated (red) flies at 10 ℃. (*E*) Sleep profile of vehicle-treated (black) or methoprene-treated (red) flies at 10℃. (*F*) Shaded sleep PI profile of vehicle-treated (black) or methoprene-treated (red) flies at 10℃

Figure S3

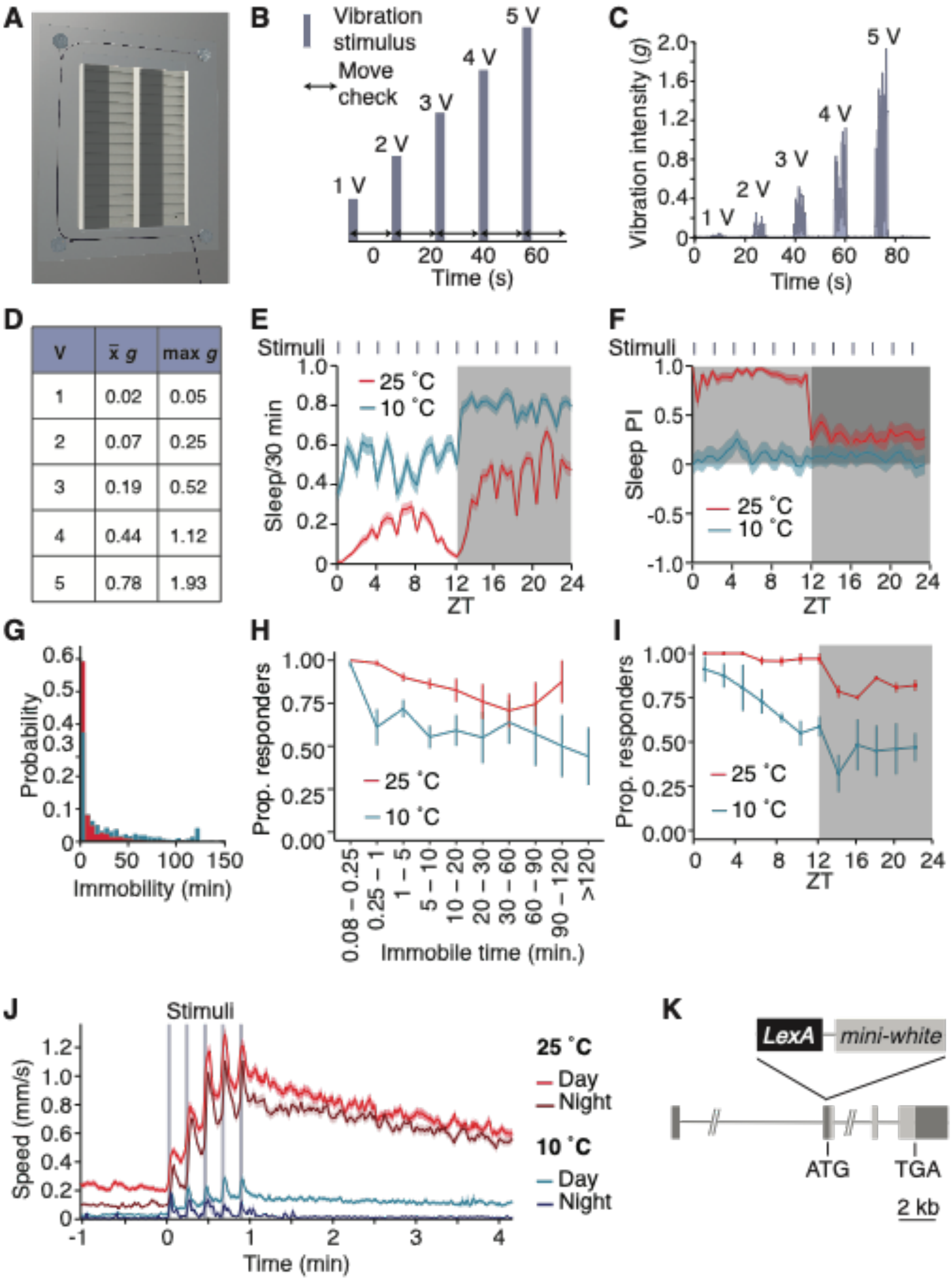
A
B
Vibration stimulus
Move check
1 V
2 V
3 V
4 V
5 V
0
20
40
60
Time (s)
C
Vibration intensity (g)
2.0
1.6
1.2
0.8
0.4
0
1 V
2 V
3 V
4 V
5 V
0
20
40
60
80
Time (s)
D
V | x̄ g | max g
1 | 0.02 | 0.05
2 | 0.07 | 0.25
3 | 0.19 | 0.52
4 | 0.44 | 1.12
5 | 0.78 | 1.93
E
Stimuli
Sleep/30 min
1.0
0.8
0.6
0.4
0.2
0
25 ˚C
10 ˚C
0
4
8
12
16
20
24
ZT
F
Stimuli
Sleep PI
1.0
0.5
0
-0.5
-1.0
25 ˚C
10 ˚C
0
4
8
12
16
20
24
ZT
G
Probability
0.6
0.5
0.4
0.3
0.2
0.1
0
0
50
100
150
Immobility (min)
H
Prop. responders
1.00
0.75
0.50
0.25
0.00
25 ˚C
10 ˚C
0.08 – 0.25
0.25 – 1
1 – 5
5 – 10
10 – 20
20 – 30
30 – 60
60 – 90
90 – 120
>120
Immobile time (min.)
I
Prop. responders
1.00
0.75
0.50
0.25
0.00
25 ˚C
10 ˚C
0
4
8
12
16
20
24
ZT
J
Stimuli
Speed (mm/s)
1.2
1.0
0.8
0.6
0.4
0.2
0
-1
0
1
2
3
4
Time (min)
25 ˚C
Day
Night
10 ˚C
Day
Night
K
LexA
mini-white
ATG
TGA
2 kb

Fig. S3. Vibration intensity and arousal threshold of flies in diapause. (*A*) Model of behavioral arena affixed with a vibrating motor at each corner. (*B*) Vibration stimulus train diagram. Every two hours, 1–5 volts were delivered to each vibrating motor, which served to increase the vibration intensity (shown in S2*C*). Each stimulus was delivered for three seconds followed by a ten second pause. Movement was scored for arousal threshold experiments when a fly that was immobile prior to the stimulus train moved at least 3 mm, either during the vibration stimulus or in the proceeding 10 seconds. (*C*) Vibration intensity (*g* force) of the behavioral arena (Fig. S3*A*) when 1–5V are delivered to the vibrating motors via pulse-width modulation from an Arduino microcontroller. (*D*) Table summarizing the vibration intensity on the arena when 1–5 volts is delivered to the four vibrating motors (Fig. S3*A*) flanking the behavioral arena. (*E*) Average sleep profile of flies at 25℃ (red) or 10℃ (blue) during arousal threshold experiments. “Stimuli” denotes the start of the stimulus train, which was delivered to flies once every two hours. Shading represents SEM. (*F*) Average sleep preference index of flies at 25℃ (red) or 10℃ (blue) during arousal threshold experiments. “Stimuli” denotes the start of the stimulus train, which was delivered to flies once every two hours. Shading represents SEM. (*G*) Histogram displaying probability of immobility length of flies housed in 25℃ (red) or 10℃ (blue) prior to experiencing stimulus train. (*H*) The proportion of flies that responded to any vibration stimulus vs. their length of immobility when maintained at 25℃ or 10℃. Line denotes the group mean and error bars denote SEM. (*I*) The proportion of flies that responded to any vibration stimulus vs. the time of the day when maintained at 25℃ or 10℃. We did not observe at 25 °C flies that were immobile for >120 min. Means ±SEMs. (*J*) Truncated version of plot shown in Fig. 3*H* to aid visualizing differences in walking speed between groups and in response to vibration stimuli. Plot displays average walking speed of flies in response to vibration stimuli at 25˚ or 10℃ during the day and night. Time 0 denotes the start of the stimulus train, with the vertical gray shading indicating each vibration stimulus. *E–F*: data recorded from 149 *w1118* flies over three independent experiments. (*K*) Cartoon depicting *rh7LexA* allele. To generate *rh7LexA* 179 bp were deleted after the translation start site in the second exon, and in its place a *LexA* and *mini-white* were inserted in frame.

Figure S4

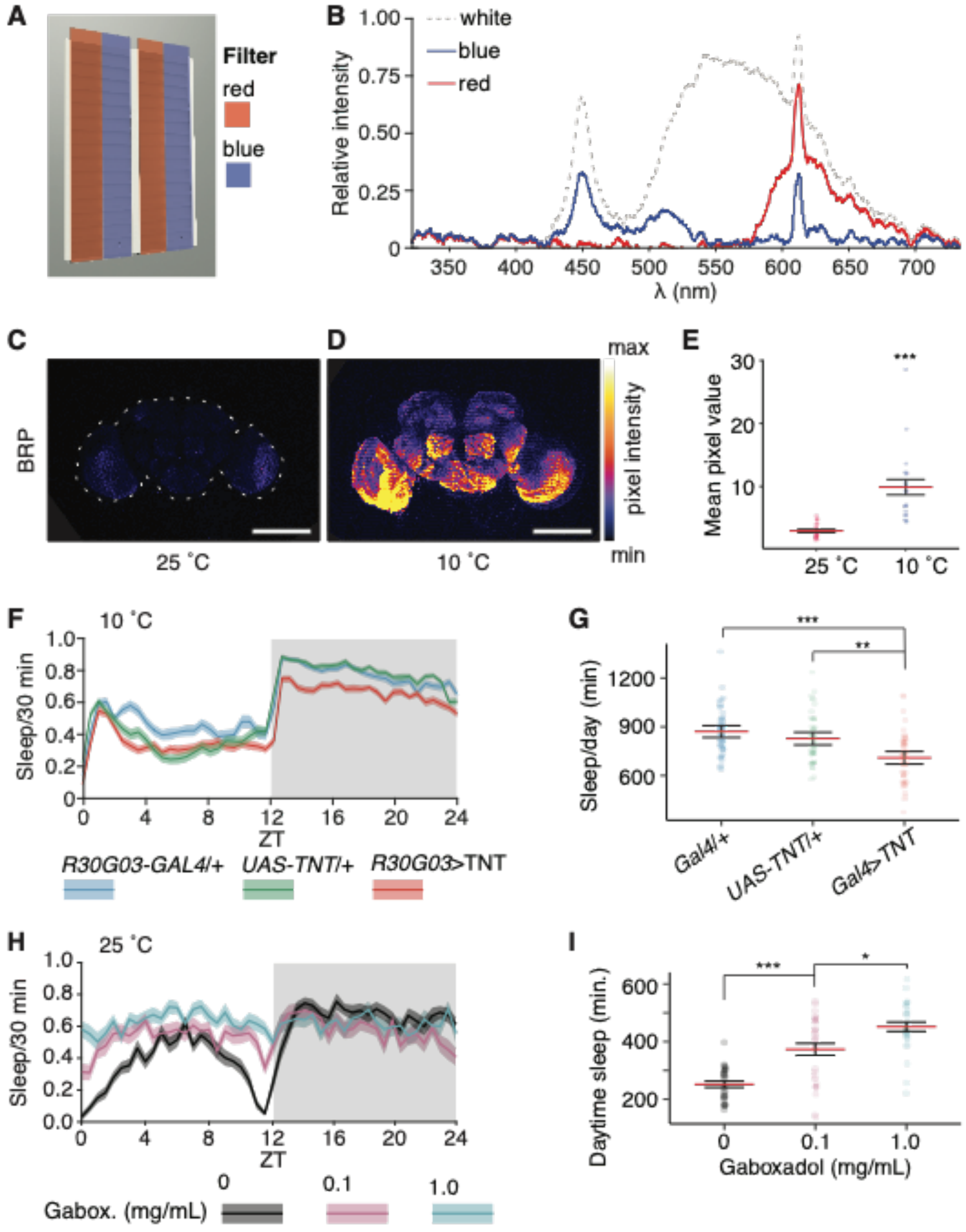

A
Filter
red
blue
B
white
blue
red
Relative intensity
λ (nm)
C
D
E
BRP
25 ˚C
10 ˚C
max
pixel intensity
min
Mean pixel value
***
F
10 ˚C
Sleep/30 min
ZT
R30G03-GAL4/+
UAS-TNT/+
R30G03>TNT
G
Sleep/day (min)
***
**
Gal4/+
UAS-TNT/+
Gal4>TNT
H
25 ˚C
Sleep/30 min
ZT
Gabox. (mg/mL)
0
0.1
1.0
I
Daytime sleep (min.)
***
*
Gaboxadol (mg/mL)

Fig. S4. Red versus blue arena, *rh7* BRP staining, and *R30G03*>TNT sleep. (*A*) Diagram of behavioral arena testing red vs. blue preference. (*B*) Relative light intensity in behavioral arena with red or blue filter. (*C*) Representative images showing BRP staining in *rh7LexA* flies maintained at 25℃ Scale bar indicates 100 μm. (*D*) Representative images showing BRP staining in *rh7LexA* flies maintained at 10℃. Outline (white dashed line) circumscribes the brain. Scale bar indicates 100 μm. (*E*) Quantification of mean pixel intensity of brains shown in B. Groups compared via Wilcoxon rank sum test. ****P*<0.001. n=21–22 brains/temperature. (*F*) Sleep profile of *R30G03*>TNT flies at 10℃. (*G*) Quantification of total sleep per day of *R30G03*>TNT shown in D. Aligned-Rank Transform ANOVA with group differences compared via Aligned Rank Transform Contrasts with Bonferroni multiple testing correction. ****P<0.001*. (*H*) Sleep profile of wild-type flies fed 0, 0.1, or 1 mg/mL of gaboxadol (Gabox.) (*I*) Total daytime sleep of wild-type flies given gaboxadol. Errorbars indicate SEM. Data compared via Aligned-Rank Transform ANOVA with group differences compared via Aligned Rank Transform Contrasts with a Bonferroni multiple testing correction.

Movie S1 (separate file). Example video displaying video tracking of Canton S. flies at 25 ˚C under white light at ZT 4 in the shade preference arena.

SI Reference

1. Q. Geissmann, L. G. Rodriguez, E. J. Beckwith, G. F. Gilestro, Rethomics: An R framework to analyse high-throughput behavioural data. *PLoS ONE* 14(1): e0209331. (2019).
